# Supplementary material for: Fully automated quantification of in vivo viscoelasticity of prostate zones using magnetic resonance elastography with Dense U-net segmentation
Source: Sci Rep. 2022 Feb 7;12:2001. doi: 10.1038/s41598-022-05878-5 (PMC8821548; doi:10.1038/s41598-022-05878-5)
Supplement: Supplementary file 1 — Supplementary Information. [file 41598_2022_5878_MOESM1_ESM.pdf]

Fully automated quantification of in vivo viscoelasticity of prostate zones using magnetic resonance elastography with Dense U-net segmentation

Nader Aldoj[1], Federico Biavati[1], Marc Dewey[1,2,3], Anja Hennemuth[4], Patrick Asbach[1] and Ingolf Sack[1]

[1]Department of Radiology, Charité – Universitätsmedizin Berlin, Berlin, Germany

[2]Berlin Institute of Health at Charité – Universitätsmedizin Berlin, Berlin, Germany

[3]DKTK (German Cancer Consortium), partner site Berlin, Germany

[4]Institute of Computer-assisted Cardiovascular Medicine, Charité – Universitätsmedizin Berlin, Berlin, Germany

Bland-Altman plots

A Bland-Altman plot depicts the degree of agreement between two separate measurements by plotting the mean vs the difference between the two measurements [1, 2].

Figures 1 and 2 show Bland-Altman graphs illustrating the levels of agreement between ground truth and predicted masks, generated by our trained networks. As shown by the plots and results in Tables 1 and 2, there is no systematic bias between quantitative values of the underlying pixels of PG, CZ and PZ regions of interest using the ground truth and the predicted masks. This was shown using the one-sample t-test between the mean difference of the observations and 0 which yielded $p>0.05$ across all segmented areas (PG, CZ and PZ) for both IMs and the UM.

Furthermore, all plots confirm a high level of agreement since most of points (we can define a prior: e.g. 90%) were within the limits of agreement (Mean±1.96 standard deviation)

Detailed results for bias (mean difference between two measures), 95% confidence intervals, and one-sample t-test with p-value between the bias and 0 are reported in Table 1 and Table 2 for IMs and the UM, respectively.

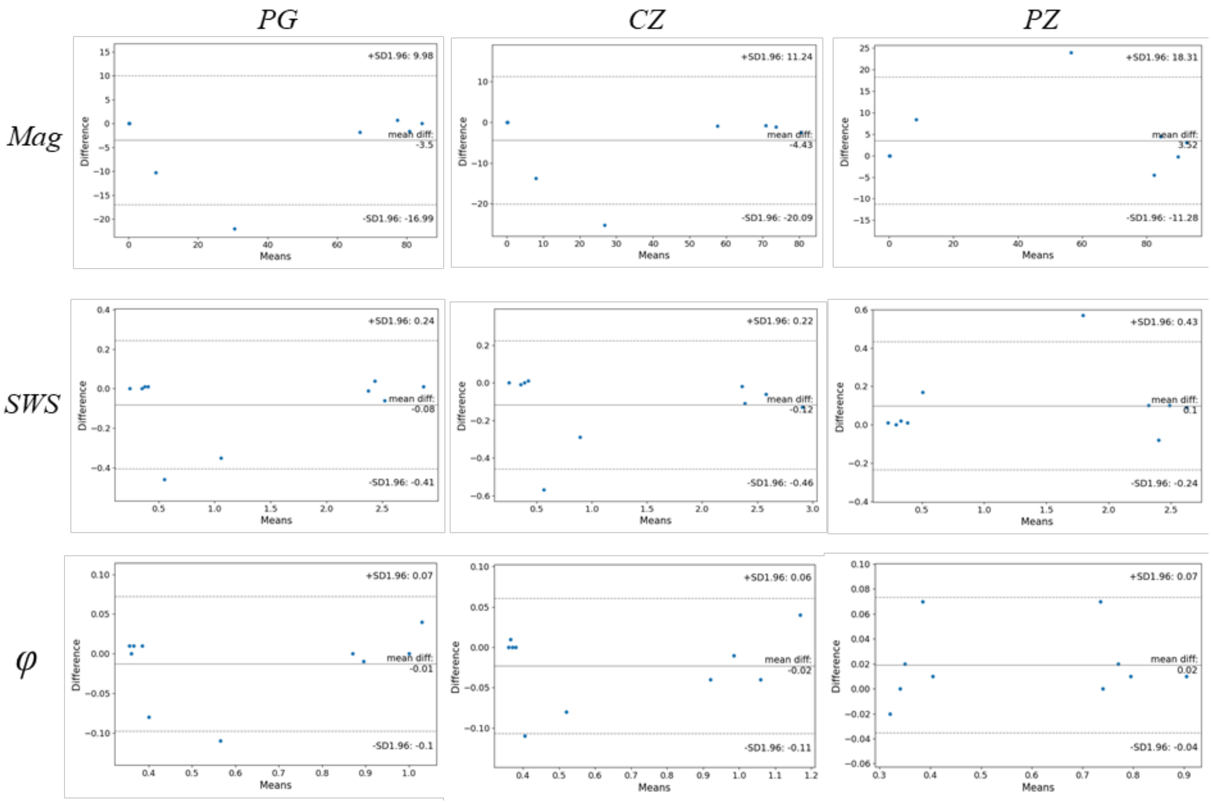

Figure s1: Bland–Altman plot between predicted and ground truth masks of all patients in the test set for both IMs models based on the resulted masks that were overlaid on SWS, mag, and φ maps. Based on segmentation results using input images of Mag map. In each of the subplots, X-axis is the mean value of the two measures. Y-axis is the difference of the two measures. Mean diff. denotes the mean difference while SD denotes the standard deviation.

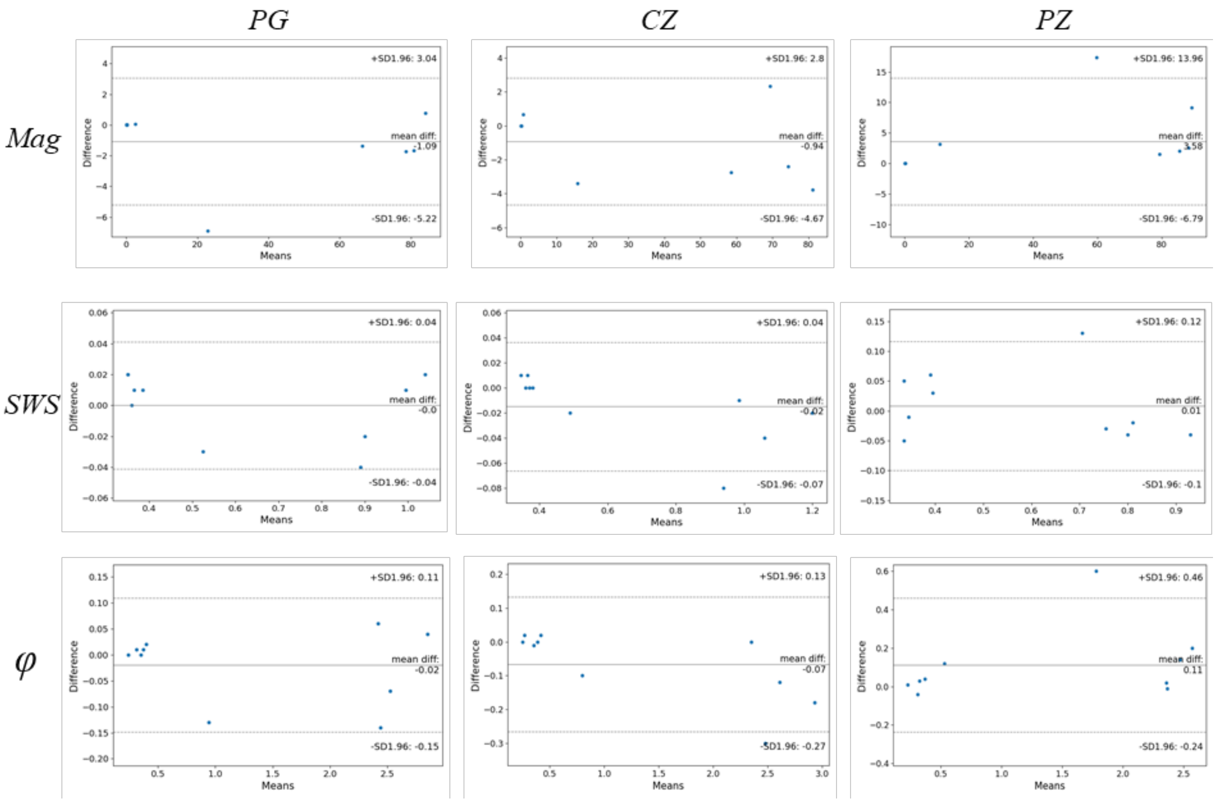

Figure s2: Bland–Altman plot between predicted and ground truth masks of all patients in the test set for both UM models based on the resulted masks that were overlaid on SWS, mag, and φ maps. Based on segmentation results using input images of Mag map. In each of the subplots, X-axis is the mean value of the two measures. Y-axis is the difference of the two measures. Mean diff. denotes the mean difference while SD denotes the standard deviation.

Table s1: Detailed statistical values of the underlying pixels of PG, CZ and PZ region of interest using ground truth original and predicted masks for all patients in the test set. Based on segmentation results from IMs using input images of Mag map. Mean diff: mean difference (known as well as Bias), SD: standard deviation, CI: confidence interval, and t-test: One-sample t-test

		Pixel values (Mean ± standard deviation)					
Map	**Patient ID**	**PG mask**	**PG predicted**	**CZ mask**	**CZ predicted**	**PZ mask**	**PZ predicted**
Mag	0	0.19±0.06	0.19±0.06	0.18±0.05	0.18±0.05	0.21±0.03	0.2±0.04
	1	0.16±0.04	0.16±0.04	0.15±0.02	0.15±0.03	0.17±0.03	0.18±0.03
	2	65.67±25.08	67.49±24.72	57.19±17.37	58.11±17.04	80.05±19.38	84.58±19.04
	3	84.45±22.7	84.43±22.8	79.28±19.44	81.8±18.6	94±12.95	90.88±13.75
	4	77.72±22.37	76.99±22.55	73.16±17.94	74.23±17.6	86.58±14.59	82.11±14.81
	5	79.94±16.3	81.61±18.54	70.47±10.33	71.22±10.6	89.71±12.9	89.93±15.44
	6	0.23±0.09	0.23±0.09	0.23±0.08	0.24±0.08	0.24±0.04	0.22±0.04
	7	0.13±0.06	0.13±0.05	0.12±0.05	0.12±0.05	0.15±0.03	0.15±0.03
	8	19.46±13.96	41.44±13.39	14.19±10.98	39.41±10.99	68.47±8.86	44.53±7.76
	9	2.65±4.05	12.96±8.32	1.08±2.27	14.86±8.09	12.59±3.36	4.23±2.02
	Mean diff. ± SD	-3.50±0.16		-4.43±8.42		-0.01±0.04	
	CI 95%	-3.50±4.49		-4.43±5.22		-0.01±0.03	
	t-test (p-value)	0.14		0.13		0.38	
SWS	0	0.24±0.08	0.24±0.08	0.25±0.07	0.25±0.07	0.23±0.04	0.22±0.04
	1	0.41±0.09	0.4±0.09	0.43±0.07	0.42±0.07	0.39±0.06	0.38±0.06
	2	2.49±0.93	2.55±0.92	2.55±0.79	2.61±0.78	2.36±0.56	2.44±0.54
	3	2.87±0.76	2.86±0.76	2.84±0.69	2.97±0.67	2.67±0.36	2.58±0.38
	4	2.37±0.68	2.38±0.7	2.33±0.58	2.44±0.59	2.37±0.39	2.27±0.4
	5	2.45±0.48	2.41±0.52	2.35±0.34	2.37±0.35	2.54±0.35	2.44±0.4
	6	0.38±0.14	0.37±0.14	0.39±0.13	0.39±0.13	0.34±0.06	0.32±0.06
	7	0.35±0.15	0.35±0.15	0.35±0.15	0.36±0.15	0.29±0.05	0.29±0.05
	8	0.88±0.45	1.23±0.32	0.75±0.37	1.04±0.22	2.08±0.26	1.51±0.23
	9	0.32±0.15	0.78±0.37	0.28±0.11	0.85±0.36	0.59±0.11	0.42±0.1
	Mean diff. ± SD	-0.08±0.17		-0.12±0.18		0.1±0.18	
	CI 95%	-0.08±0.11		-0.12±0.11		0.1±0.11	
	t-test (p-value)	0.17		0.07		0.11	
φ	0	0.37±0.12	0.36±0.12	0.37±0.11	0.37±0.11	0.34±0.06	0.34±0.06
	1	0.36±0.08	0.35±0.08	0.37±0.06	0.36±0.07	0.36±0.05	0.34±0.05
	2	0.89±0.35	0.9±0.34	0.98±0.31	0.99±0.3	0.74±0.18	0.74±0.17
	3	1±0.28	1±0.28	1.04±0.26	1.08±0.25	0.8±0.11	0.79±0.12
	4	0.87±0.26	0.87±0.27	0.9±0.23	0.94±0.23	0.78±0.13	0.76±0.14
	5	1.05±0.22	1.01±0.24	1.19±0.18	1.15±0.18	0.91±0.13	0.9±0.16
	6	0.39±0.15	0.38±0.15	0.38±0.13	0.38±0.13	0.41±0.08	0.4±0.07
	7	0.36±0.16	0.36±0.16	0.36±0.16	0.36±0.15	0.31±0.05	0.33±0.06
	8	0.51±0.2	0.62±0.15	0.48±0.18	0.56±0.11	0.77±0.09	0.7±0.1
	9	0.36±0.11	0.44±0.17	0.35±0.1	0.46±0.16	0.42±0.05	0.35±0.06
	Mean diff. ± SD	-0.01±0.04		-0.02±0.04		0.02±0.03	
	CI 95%	-0.01±0.03		-0.02±0.03		0.02±0.02	
	t-test (p-value)	0.39		0.14		0.07	

Table s2: Detailed statistical values of the underlying pixels of PG, CZ and PZ region of interest using ground truth original and predicted masks for all patients in the test set. Based on segmentation results from **UM** using input images of Mag map. Mean diff: mean difference (known as well as Bias), SD: standard deviation, CI: confidence interval, and t-test: One-sample t-test

		Pixel values (Mean ± standard deviation)					
Map	**Patient ID**	**PG mask**	**PG predicted**	**CZ mask**	**CZ predicted**	**PZ mask**	**PZ predicted**
Mag	0	0.19±0.06	0.18±0.06	0.18±0.05	0.18±0.05	0.21±0.03	0.19±0.04
	1	0.16±0.04	0.16±0.04	0.15±0.02	0.15±0.03	0.17±0.03	0.17±0.03
	2	65.67±25.08	67.05±24.22	57.19±17.37	59.94±17.05	80.05±19.38	78.53±18.29
	3	84.45±22.7	83.68±22.98	79.28±19.44	83.05±18.32	94±12.95	84.83±14.47
	4	77.72±22.37	79.46±20.38	73.16±17.94	75.57±14.62	86.58±14.59	84.52±14.67
	5	79.94±16.3	81.62±17.95	70.47±10.33	68.14±7.99	89.71±12.9	87.18±16.22
	6	0.23±0.09	0.23±0.09	0.23±0.08	0.24±0.08	0.24±0.04	0.22±0.04
	7	0.13±0.06	0.12±0.05	0.12±0.05	0.12±0.05	0.15±0.03	0.13±0.03
	8	19.46±13.96	26.38±16.63	14.19±10.98	17.6±10.96	68.47±8.86	51.1±12.73
	9	2.65±4.05	2.59±4.14	1.08±2.27	0.42±1.17	12.59±3.36	9.45±3.97
	Mean diff. ± SD	-1.09±2.22		-0.94±2.01		3.58±5.58	
	CI 95%	-1.09±1.37		-0.94±1.24		3.85±3.46	
	t-test (p-value)	0.15		0.17		0.07	
SWS	0	0.24±0.08	0.24±0.08	0.25±0.07	0.25±0.07	0.23±0.04	0.22±0.04
	1	0.41±0.09	0.39±0.09	0.43±0.07	0.41±0.08	0.39±0.06	0.35±0.05
	2	2.49±0.93	2.56±0.91	2.55±0.79	2.67±0.76	2.36±0.56	2.37±0.54
	3	2.87±0.76	2.83±0.76	2.84±0.69	3.02±0.65	2.67±0.36	2.47±0.41
	4	2.37±0.68	2.51±0.64	2.33±0.58	2.63±0.51	2.37±0.39	2.35±0.4
	5	2.45±0.48	2.39±0.5	2.35±0.34	2.35±0.27	2.54±0.35	2.4±0.43
	6	0.38±0.14	0.37±0.14	0.39±0.13	0.39±0.13	0.34±0.06	0.31±0.06
	7	0.35±0.15	0.35±0.15	0.35±0.15	0.36±0.14	0.29±0.05	0.33±0.07
	8	0.88±0.45	1.01±0.49	0.75±0.37	0.85±0.37	2.08±0.26	1.48±0.33
	9	0.32±0.15	0.31±0.16	0.28±0.11	0.26±0.08	0.59±0.11	0.47±0.13
	Mean diff. ± SD	-0.02±0.07		-0.07±0.11		0.11±0.19	
	CI 95%	-0.02±0.04		-0.07±0.07		0.11±0.12	
	t-test (p-value)	0.38		0.08		0.09	
φ	0	0.37±0.12	0.36±0.12	0.37±0.11	0.37±0.1	0.34±0.06	0.35±0.07
	1	0.36±0.08	0.34±0.08	0.37±0.06	0.36±0.07	0.36±0.05	0.31±0.05
	2	0.89±0.35	0.91±0.34	0.98±0.31	0.99±0.29	0.74±0.18	0.77±0.18
	3	1±0.28	0.99±0.28	1.04±0.26	1.08±0.24	0.8±0.11	0.82±0.14
	4	0.87±0.26	0.91±0.24	0.9±0.23	0.98±0.2	0.78±0.13	0.82±0.15
	5	1.05±0.22	1.03±0.23	1.19±0.18	1.21±0.15	0.91±0.13	0.95±0.18
	6	0.39±0.15	0.38±0.15	0.38±0.13	0.38±0.13	0.41±0.08	0.38±0.07
	7	0.36±0.16	0.36±0.15	0.36±0.16	0.36±0.14	0.31±0.05	0.36±0.08
	8	0.51±0.2	0.54±0.22	0.48±0.18	0.5±0.18	0.77±0.09	0.64±0.13
	9	0.36±0.11	0.34±0.12	0.35±0.1	0.34±0.1	0.42±0.05	0.36±0.07
	Mean diff. ± SD	0.00±0.02		-0.01±0.03		0.01±0.06	
	CI 95%	0.00±0.01		-0.01±0.02		0.01±0.04	
	t-test (p-value)	1		0.12		0.67	

References

1. Bland, J.M. and D. Altman, *Statistical methods for assessing agreement between two methods of clinical measurement.* The lancet, 1986. **327**(8476): p. 307-310.
2. Bland, J.M. and D.G. Altman, *Measuring agreement in method comparison studies.* Statistical methods in medical research, 1999. **8**(2): p. 135-160.
